# Diabetes aggravates myocardial ischaemia reperfusion injury via activating Nox2‐related programmed cell death in an AMPK‐dependent manner

**DOI:** 10.1111/jcmm.15318

**Published:** 2020-04-29

**Authors:** Chunyan Wang, Lijie Zhu, Wenlin Yuan, Lingbin Sun, Zhengyuan Xia, Zhongjun Zhang, Weifeng Yao

**Affiliations:** ^1^ Department of Anesthesiology Shenzhen People's Hospital and Shenzhen Anesthesiology Engineering Center The Second Clinical Medical College of Jinan University Shenzhen China; ^2^ Department of Pathophysiology School of Medicine Shenzhen University Shenzhen China; ^3^ Department of Anesthesiology Li Ka Shing Faculty of Medicine The University of Hong Kong Hong Kong China; ^4^ Department of Anesthesiology The Third Affiliated Hospital of Sun Yat‐sen University Guangzhou China

**Keywords:** AMPK, diabetes, myocardial ischaemia reperfusion injury, Nox2, programmed cell death

## Abstract

Cardiovascular diseases such as myocardial ischaemia have a high fatality rate in patients with diabetes. This study was designed to expose the crosstalk between oxidative stress and AMPK, a vital molecule that controls biological energy metabolism, in myocardial ischaemia reperfusion injury (I/RI) in diabetic rats. Diabetes was stimulated in rats using streptozotocin injection. Rats were separated on random into control, control + I/R, Diabetes, Diabetes + I/R, Diabetes + I/R + N‐acetylcysteine and Diabetes + I/R + Vas2870 groups. Myocardial infarct size was determined, and the predominant Nox family isoforms were analysed. In vitro, the H9C2 cells were administered excess glucose and exposed to hypoxia/reoxygenation to mimic diabetes and I/R. The AMPK siRNA or AICAR was used to inhibit or activate AMPK expression in H9C2 cells, respectively. Then, myocardial oxidative stress and programmed cell death were measured. Diabetes or high glucose levels were found to aggravate myocardial I/RI or hypoxia/reoxygenation in H9C2 cells, as demonstrated by an increase in myocardial infarct size or lactate dehydrogenase levels, oxidative stress generation and induction of programmed cell death. In diabetic rat hearts, cardiac Nox1, Nox2 and Nox4 were all heightened. The suppression of Nox2 expression using Vas2870 or Nox2‐siRNA treatment in vivo or in vitro*,* respectively, protected diabetic rats from myocardial I/RI. AMPK gene knockout increased Nox2 protein expression while AMPK agonist decreased Nox2 expression. Therefore, diabetes aggravates myocardial I/RI by generating of Nox2‐associated oxidative stress in an AMPK‐dependent manner, which led to the induction of programmed cell death such as apoptosis, pyroptosis and ferroptosis.

## 1 INTRODUCTION

Myocardial ischaemia reperfusion injury (I/RI) develops in patients with diabetes during surgery, which leads to an increase in the incidence of complications and mortality that can have serious consequences. Several studies have shown that approximately 10% of patients with diabetes and ischaemic heart disease can develop cardiovascular complications, and among 0.65% of such patients, the complications can prove fatal. The myocardium has greater sensitivity to I/RI in those with diabetes, which is accompanies by a worse prognosis.^1^, ^2^

The creation of reactive oxygen species (ROS) and subsequent mitochondrial injury are among the most significant causal factors that are mechanistically associated with myocardial I/RI. Nicotinamide adenine dinucleotide phosphate (NADPH) oxidase (Nox) is the main donor of ROS amidst myocardial I/RI.^3^, ^4^ There are mainly three subtypes of Nox in the cardiomyocytes, Nox1, Nox2 and Nox4. Previous studies have shown that Nox2 and Nox4 can induce a large amount of ROS generation and have a significant function in causing injury in various heart disease models.^5^, ^6^, ^7^ However, the role of Nox enzymes and the Nox subtypes in the diabetic myocardium is still unclear.

Myocardial I/RI often cause myocardial cell death. Apoptosis, pyroptosis and ferroptosis are the main forms of myocardial programmed cell death.^8^, ^9^ Recent studies have confirmed that these three types of programmed cell death pathways exist in I/RI. Studies indicate that apoptosis is involved in many animal models that suffer from myocardial I/RI.^10^, ^11^ Blocking of pyroptosis could substantially reduce the myocardial infarction area. The influence of ferroptosis on myocardial I/RI is inconsistent. However, the occurrence and development of these three types of programmed cell death in myocardial I/RI are not fully understood, and whether they play an important function in diabetic myocardial I/RI is not clear.

Therefore, this study aims to identify the underlying mechanism associated with diabetic myocardial I/RI and examines the role of NADPH enzyme‐related oxidative stress as well as myocardial cell loss in diabetic myocardial I/RI.

## 2 MATERIAL AND METHODS

### 2.1 Animal and stimulation of diabetes

Male Sprague Dawley rats (250 g), aged 6‐8 weeks, were acquired through the Guangdong Medical Lab Animal Center. Diabetes was stimulated by administering one dosage (65 mg/kg) of streptozotocin (STZ) (Sigma‐Aldrich) through tail vein inoculation. N‐acetylcysteine (NAC) (1.5 g/kg/d) was added to the rats' water for 8 weeks starting 1 week post diabetes induction, but prior to the development ischaemia reperfusion (I/R) damage. Rats were placed in the Laboratory Animal Service Center of Jinan University, where they were administered standard care using a 12‐hour dark/light cycle and were given food/water as per guidelines established by Animal Care of Jinan University.

### 2.2 Animal experimental protocol

Animals were separated, on random, into six groups: controls (Con), control + I/R (Con + I/R), Diabetes (D), Diabetes + I/R (D + I/R), Diabetes + I/R + NAC (D + I/R + NAC) and Diabetes + I/R + Vas2870 (D + I/R + Vas287). Vas2870 (2 mg/kg) was administered through the jugular vein 10 minutes prior to inducing I/R. The rats were anaesthetized by administering phenobarbital sodium (50 mg/kg). Ischaemia was attained through blocking the left anterior descending (LAD) artery for 30 minutes and subsequently underwent reperfusion for 2 hours, as previously shown.^12^, ^13^ Finally, rats were placed under euthanasia by injecting a high dose of pentobarbital. Additionally, 5% Evans Blue was administered via the right jugular vein to mark the healthy area of left ventricle^2^ and to measure the anatomic area at risk (AAR). Hearts were extracted and divided in 5 slices of 1 mm cross section. Then, they were placed in incubation with 1% 2, 3, 5‐triphenyltetrazolium chloride (TTC) in phosphate‐buffered saline (PBS) for 20 minutes at room temperature to determine infarct size (IS), which was represented as proportion of AAR. Slices were fixed using 10% formalin. Regions of left ventricular (LV) tissue were rapidly frozen using liquid nitrogen. Samples underwent additional processing for Western blot.

### 2.3 Detecting apoptotic cell death utilizing terminal deoxynucleotidyl transferase dUTP nick‐end labelling (TUNEL)

TUNEL staining was conducted to identify apoptosis as per established guidelines (Roche Applied Science). In brief, LV tissue was fixed with 4% formalin, embedded in paraffin, divided (5 mm thick sections) and de‐paraffinized. Then, sections underwent permeabilization utilizing proteinase K (30 mg/mL, 30 minutes, 37°C), followed by washing with PBS. DNase I helps stimulate DNA strand breaks as positive control, and TdT was removed from reaction mixture as the negative control. Fluorescence was observed under a fluorescent microscope (Leica, Dmi8 + DFC 7000T) at an absorption spectrum of 488 nm and an emission spectrum of 530 nm.

### 2.4 Immunohistochemical assay for 4‐hydroxynonenal (4‐HNE)

Immunohistochemical staining was performed to identify 4‐HNE in the paraffin‐embedded heart sections using the anti‐4‐HNE antibody (1:100; ab46545, Abcam), and fluorescence was observed under a fluorescent microscope (Leica, Dmi8 + DFC 7000T).

### 2.5 Nox2 and AMPK siRNA studies in H9C2 cells

The rat cardiac H9C2 cells were grown in Dulbecco's modified Eagle's medium (DMEM) supplemented with 10% FBS at 5% CO_2_ and 37°C. In order to simulate the diabetic conditions of high glucose (HG), d‐glucose (25 mmol/L) was supplemented into the medium, which led to a total glucose dose of 30 mmol/L. Administration of normal glucose concentration (5.5 mmol/L) in the medium was utilized as a control. Lastly, 19.5 mmol/L mannitol and 5.5 mmol/L glucose were supplemented for osmotic control.

In order to validate function of AMPK and Nox2, gene silencing was conducted utilizing transfection reagent and siRNAs against AMPK, Nox2 and control (Santa Cruz Biotechnology, Dallas, USA). Briefly, 2 * 10^6^ H9C2 cells were placed in culture with DMEM encompassing 10% FBS at 5% CO_2_ and 37°C. Cells were transfected using targeted and control siRNA once they reached 60%‐80% confluence. Medium was removed 6 hours after transfection, fresh medium was added and cells were subjected to normal or HG medium for 48 hours. Next, the cells were exposed to hypoxia for 6 hours and reoxygenation for 12 hours either with or without excess glucose (30 mmol/L). Hypoxia was achieved in an incubator with 1% O_2,_ 5% CO_2_ and 94% N_2_. Reoxygenation was attained by exposure of cells to CO_2_ incubator.

### 2.6 Determination of lactate dehydrogenase (LDH), Troponin‐T (TN‐T), malonaldehyde (MDA) and Superoxide Dismutase (SOD) levels

TN‐T is the gold standard for definition of myocardial I/RI, while MDA release and SOD activity levels are standard oxidative indices. At the end of reperfusion, blood samples were gathered by carotid artery and spun down to divide the serum for detection of TN‐T, MDA and SOD levels utilizing their respective ELISA kits from Shang Hai Jiang Lai biological following the manufacturer's instructions. For H9C2 cells, post‐H/R, the medium was gathered and spun down to detect LDH, MDA and SOD levels using the LDH cytotoxicity assay kit (Roche, USA), MDA and SODELISA kit from Shang Hai Jiang Lai biological.

### 2.7 Western blot

Frozen ventricular tissue was emulsified in RIPA buffer (Cell Signaling Technology) and spun down at 13 200 *g *for 30 minutes at 4°C. Similarly, H9C2 cells were added to the lysis buffer and spun down at 13 200 *g* for 15 minutes at 4°C. The protein concentration was evaluated by utilizing the Bradford assay. Equivalent quantities of proteins from both H9C2 cells and rat hearts were ran on 7.5%‐12.5% SDS‐PAGE gel. Next, protein was transferred onto polyvinylidene nitrocellulose (PVDF) membranes, which was then placed in blocking buffer (TBST with 5% (w/v) non‐fat milk) for 1 hour at room temperature. Membranes were incubated with primary antibodies at 4°C overnight. The primary antibodies against Nox1 (NOVUS), Nox2 (Abcam), Nox4 (Abcam), AMPK (Cell Signaling Technology, Inc), phospho‐AMPK (Cell Signaling Technology, Inc), GPX4 (Thermo Fisher Scientific, Inc), NLRP3 (Abcam), cleaved caspase‐3 (Cell Signaling Technology, Inc) and GAPDH (Cell Signaling Technology, Inc). After primary incubation, the membranes were washed 3 times for 10 minutes every time. Membranes were incubated using either anti‐rabbit or anti‐mouse IgG secondary antibody (1:10 000; Cell Signaling Technology, Inc) for 1 hour. The proteins were identified using a conventional ECL method. The bands were quantified utilizing a densitometer.

### 2.8 Statistical analyses

Data is represented as mean ± standard error of mean (SEM). The data were normally distributed according to the GraphPad Prism normality test. The comparison of many groups and in vivo treatments was evaluated using one‐way ANOVA, which was followed by Tukey's test for multiple comparisons (GraphPad Software, Inc). *P* < 0.05 represents statistical significance.

## 3 RESULTS

### 3.1 Increase in myocardial oxidative stress and programmed cell death after myocardial I/RI in diabetic hearts

**FIGURE 1:**
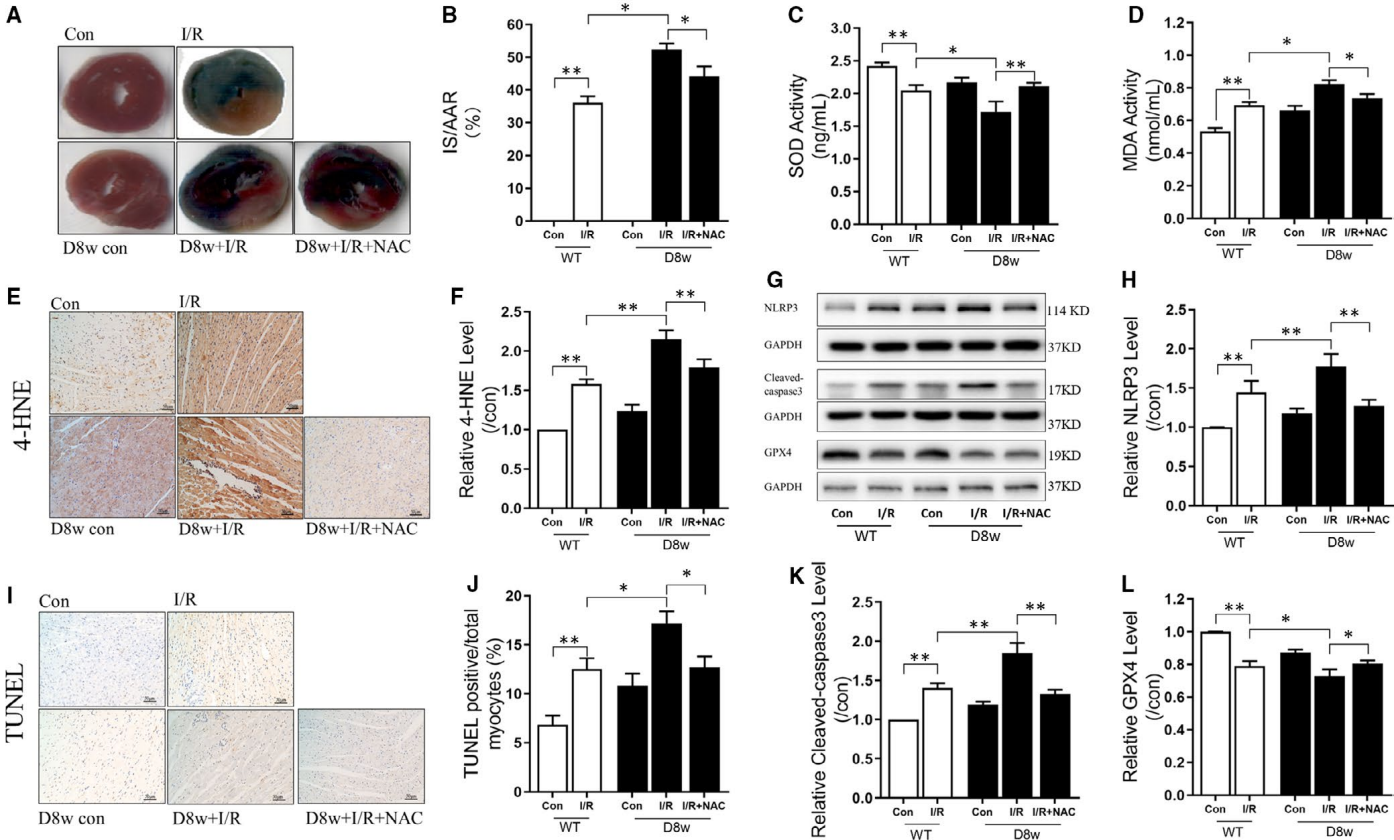
Increase in myocardial oxidative stress and programmed cell death after myocardial I/R injury in diabetic hearts. A, The infarct size (IS) in the Con and diabetic rats determined by TTC and Evans blue staining; B, Post‐ischaemia IS expressed as percentage of IS to the area at risk (AAR); C, SOD activity during myocardial I/RI in Con and diabetic rats; D, MDA release during myocardial I/RI in Con and diabetic rats; E, 4‐HNE staining; F, Changes in relative 4‐HNE levels in Con and diabetic rats; G, The WB bands showing the protein expression of NLRP3, Cleaved caspase‐3, GPX4 and GAPDH; H, The change in protein expression levels of NLRP3 during myocardial I/RI in Con and diabetic rats; I, TUNEL staining; J, TUNEL positive/total myocytes; K, the change in protein expression levels of cleaved caspase‐3 in Con and diabetic rats; L, The change in protein expression levels of GPX4 during myocardial I/RI in Con and diabetic rats. Myocardial ischaemia reperfusion (I/R) was achieved by 30 min ischaemia and 120 min reperfusion. Data are expressed as mean ± SEM, n = 6 per group. *P < 0.05, **P < 0.01

As demonstrated in Figure 1, in non‐diabetic hearts, post‐ischaemic IS was substantially higher after myocardial I/RI (Figure 1A,B, *P* < 0.05, Con + I/R vs Con), correlated to increase in cardiac oxidative stress as demonstrated by decreased SOD activity (Figure 1C, *P* < 0.05, Con + I/R vs Con), heightened MDA formation (Figure 1D, *P* < 0.05, Con + I/R vs Con) and increased 4‐HNE expression (Figure 1F, *P* < 0.05, Con + I/R vs Con). Additionally, we observed an induction of programmed cell death as validated by increased NLRP3 protein (pyroptosis) (Figure 1H, *P* < 0.05, Con + I/R vs Con), presence of TUNEL‐positive cells (apoptosis) (Figure 1J, *P* < 0.05, Con + I/R vs Con), enhanced cleaved caspase‐3 levels (apoptosis) (Figure 1K, *P* < 0.05, Con + I/R vs Con) and decreased expression of GPX4 (ferroptosis) (Figure 1L, *P* < 0.05, Con + I/R vs Con). Myocardial I/RI was critical in diabetic hearts (all *P* < 0.05, D8w + I/R vs Con + I/R). Interestingly, cardiac injury and programmed cell death after myocardial I/R damage in diabetic hearts were attenuated using antioxidant treatment with NAC (*P* < 0.05, D8w + I/R + NAC vs D8w + I/R). These findings demonstrate that oxidative stress can have a vital function in inducing programmed cell death and subsequent myocardial I/RI in diabetic hearts.

### 3.2 Nox blockage attenuates post‐ischaemic programmed cell death and cardiac injury in diabetic hearts

**FIGURE 2:**
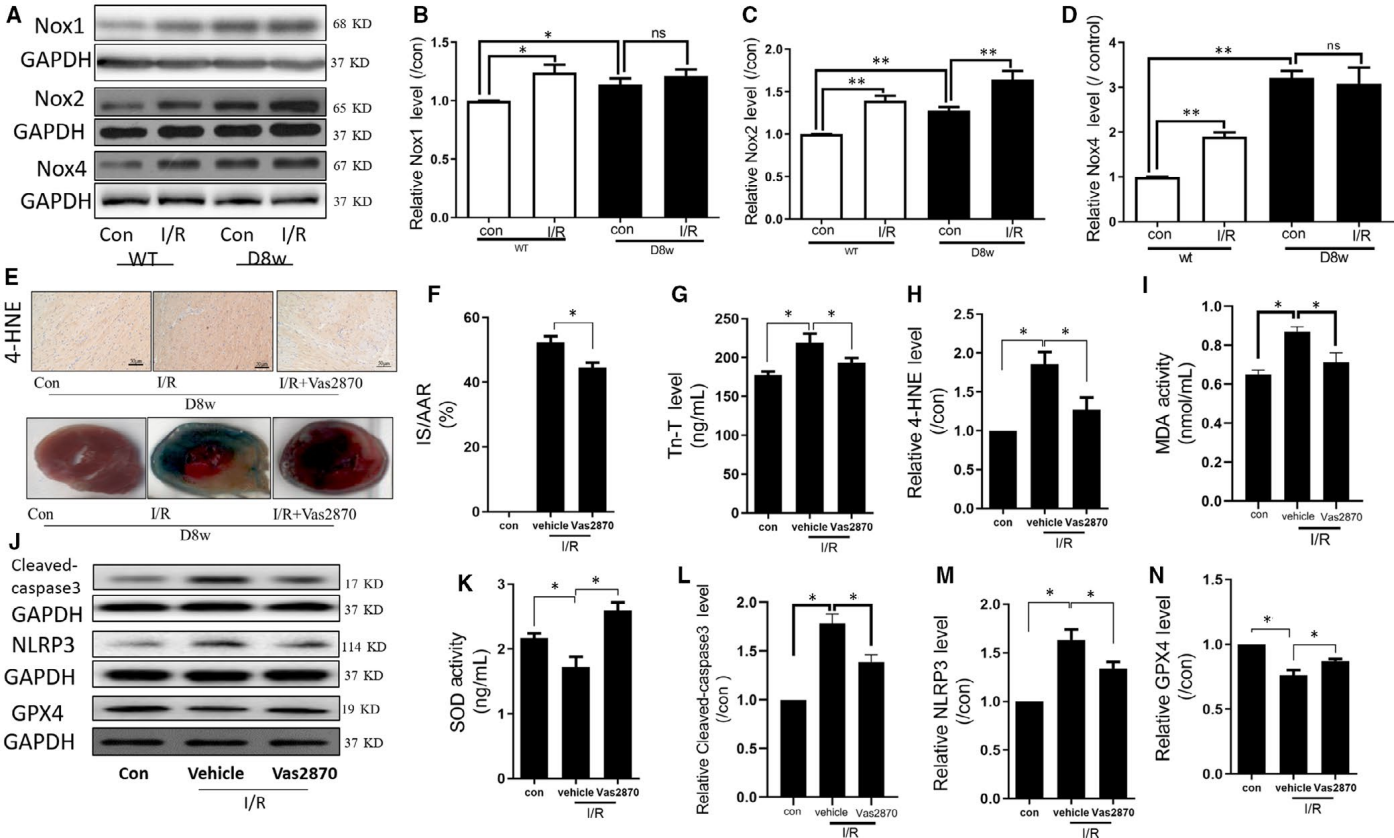
Nox inhibition attenuates post‐ischaemic programmed cell death and cardiac injury in diabetic rats. A, WB bands depicting the protein expression of Nox1, Nox2, Nox4 and GAPDH; B, The change in the protein expression of Nox1 in Con and diabetic rats; C, The change in protein expression of Nox2 in Con and diabetic rats; D, The change in protein expression of Nox4 in Con and diabetic rats; E, 4‐HNE and TTC staining; F, The change of IS in response to treatment with or without Vas2870 in diabetic rats; G, Tn‐T release during myocardial I/RI in response to treatment with or without Vas2870 in diabetic rats; H, The relative 4‐HNE levels during myocardial I/RI in diabetic rats, with or without Vas2870 treatment; I, MDA release during myocardial I/RI in diabetic rats, with or without Vas2870 treatment; J, WB bands depicting protein expression of cleaved caspase‐3, NLRP3 and GPX4; K, SOD release during myocardial I/RI in diabetic rats, with or without Vas2870 treatment; L, The change in the protein expression of cleaved caspase‐3 during myocardial I/RI in diabetic rats, with or without Vas2870 treatment; M, The change in the protein expression of NLRP3 during myocardial I/RI in diabetic rats, with or without Vas2870 treatment; N, The change in protein expression of GPX4 during myocardial I/RI in diabetic rats, with or without VAS2870 treatment. Myocardial ischaemia reperfusion(I/R) was achieved by 30 min ischaemia and 120 min reperfusion. Data are expressed as mean ± SEM, n = 6 per group. *P < 0.05, **P < 0.01

Nox1, Nox2 and Nox4 were the predominant isoforms of Nox family with functions in the heart.^14^ As shown in Figure 2A‐D, in the non‐diabetic hearts, Nox1, Nox2 and Nox4 were heightened post‐myocardial I/RI (all *P* < 0.01, Con + I/R vs Con). However, only Nox2 was further increased after myocardial I/RI in the diabetic hearts (Figure 2B‐D, *P* < 0.01, D8w + I/R vs D8w), suggesting an important function for Nox2 in myocardial I/RI in diabetic conditions. Thus, in the subsequent experiments, we focused on examining the role of Nox2 in myocardial I/RI in diabetic hearts.

To determine Nox2 function in myocardial I/R damage in diabetic hearts, diabetes was induced for 8 weeks in rats. Some diabetic rats were administered the Nox2 inhibitor Vas2870. As demonstrated in Figure 2F, post‐ischaemic IS was significantly increased (*P* < 0.05, D8w + I/R vs D8w) and was associated with an increase in the cardiac injury marker Tn‐T (Figure 2G), and post‐ischaemic myocardial oxidative stress as shown by increase in 4‐HNE expression (Figure 2H), elevated MDA levels (Figure 2I) and decreased SOD activity (Figure 2K). There was also a significant increase in the apoptotic protein cleaved caspase‐3 (Figure 2L), increase in pyroptosis protein NLRP3 (Figure 2M) and reduction in GPX4 levels (Figure 2N), the latter of which represents ferroptosis induction (all *P* < 0.05, D8w + I/R vs D8w). Furthermore, the inhibition of Nox with Vas2870 significantly reduced the injury stimulated by myocardial I/R (all *P* < 0.05, D8w + I/R + Vas2870 vs D8w + I/R). These data indicate that Nox has a vital function in myocardial I/RI in diabetic hearts.

### 3.3 Nox2 gene knockdown attenuated post‐hypoxic oxidative stress and programmed cell death in cardiomyocytes administered high glucose

**FIGURE 3:**
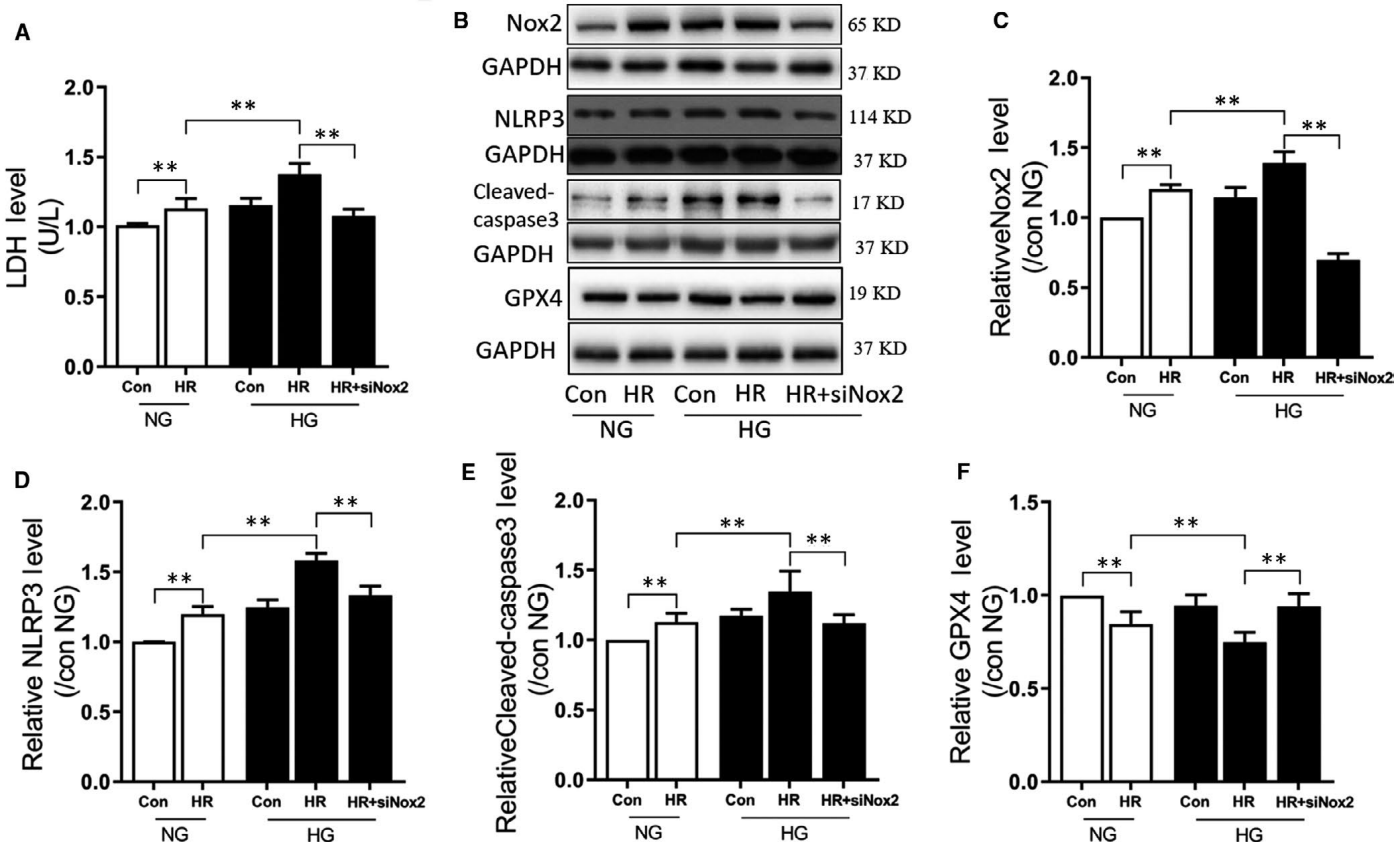
Nox2 gene knockdown attenuated post‐hypoxic oxidative stress and programmed cell death in cardiomyocytes exposed to high glucose. A, LDH release during hypoxia reoxygenation (H/R) in normal (NG) and (HG), with or without siRNA‐mediated knockdown of Nox2; B, WB band showing protein expression of Nox2, NLRP3, Cleaved caspase‐3, GPX4 and GAPDH; C, the change in protein expression of Nox2 during H/R in NG and HG, with or without siRNA Nox2; D, The change in protein expression of NLRP3 during H/R in NG and HG, with or without siRNA Nox2; E, The change in protein expression of cleaved caspase‐3 during H/R in NG and HG, with or without siRNA Nox2; F, The change in protein expression of GPX4 during H/R in NG and HG, with or without siRNA Nox2. In the HG group, H9C2 cells were subjected 30 mmol/L glucose treatment for 48 h, H/R was achieved by 6 h hypoxia exposure and 12 h reperfusion. Data are expressed as mean ± SEM of two independent experiments, each performed in triplicates. n = 6 per group. *P < 0.05, **P < 0.01

To confirm the function of Nox2 in cardiac injury damage of diabetic hearts, rat cardiomyocyte H9C2 cells were administered high glucose and hypoxia reoxygenation (H/R). As depicted in Figure 3, after H9C2 cells were administered high glucose and H/R, their cellular injury increased significantly, as manifested by the release of LDH (Figure 3A). This was correlated to increased Nox2 protein expression (Figure 3B), induction of cell pyroptosis (increased NLRP3 protein expression, Figure 3D), cell apoptosis (enhanced cleaved caspase‐3 protein levels, Figure 3E) and ferroptosis (decreased GPX4 protein expression) (Figure 3F, all *P* < 0.05, NG + H/R vs NG, HG + H/R vs NG + H/R). Additionally, the injury was reduced by Nox2 gene knockdown (all *P* < 0.05, HG + H/R + siRNA Nox2 vs HG + H/R). These data indicate the cardioprotective function of Nox2 in myocardial I/RI in the diabetic hearts.

### 3.4 AMPK works upstream of Nox2 in diabetic hearts

**FIGURE 4:**
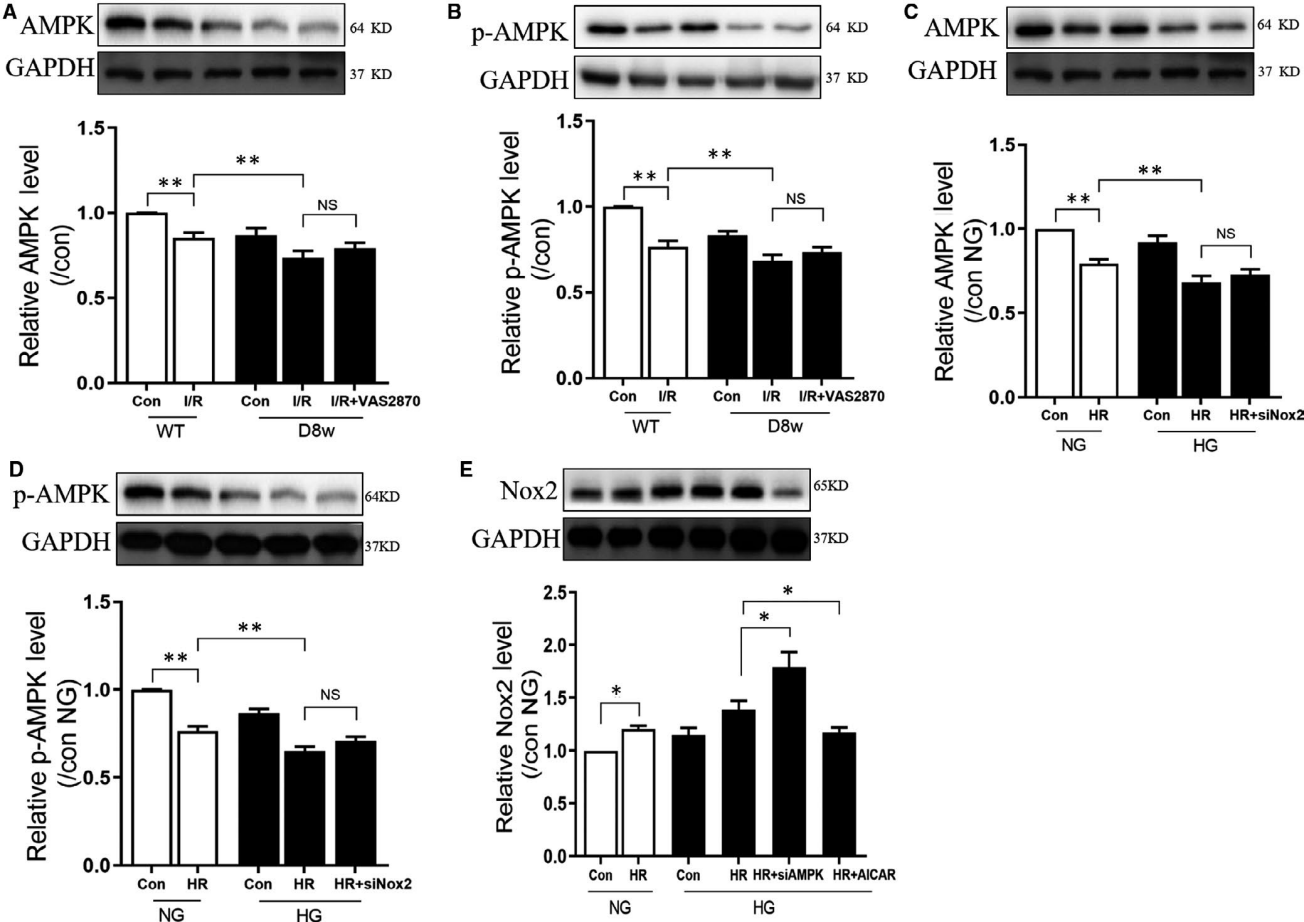
AMPK works upstream of Nox2 in diabetic hearts. A, The change in protein expression of AMPK during myocardial I/RI in Con and diabetic rats, with or without Vas2870 treatment; B, The change in protein expression of p‐AMPK during myocardial I/RI in Con and diabetic rats, with or without Vas2870 treatment; C, The change in protein expression of AMPK during H/R in NG and HG, with or without siRNA Nox2; D, The change in protein expression of p‐AMPK during H/R in NG and HG, with or without siRNA Nox2; E, The change in protein expression of p‐AMPK during H/R in NG and HG, with or without siRNA AMPK, or agonist AICAR. Myocardial ischaemia reperfusion(I/R) was achieved by 30 min ischaemia and 120 min reperfusion; in the HG group, H9C2 cells were subjected 30 mmol/L glucose treatment for 48 h, H/R was achieved by 6 h hypoxia exposure and 12 h reperfusion. Data are expressed as mean ± SEM of two independent experiments, each performed in triplicates. n = 6 per group. * *P* < 0.05, **P < 0.01

AMPK is a cardiac energy sensor, and the cardioprotective role of AMPK in diabetic hearts has previously been shown.^15^, ^16^ To evaluate the function of Nox2 in AMPK‐regulated cardio‐protection in diabetic hearts, AMPK activation (phosphorylation, p‐AMPK) was examined in the animal model system. As indicated in Figure 4A,B, in post‐ischaemic non‐diabetic rats, total protein and phosphorylation expression levels of AMPK were significantly decreased (*P* < 0.01, Con + H/R vs Con). In diabetic rats, the total and phosphorylation levels of AMPK were further decreased by myocardial I/RI (*P* < 0.01, D8w + H/R vs Con + H/R). Inhibition of Nox2 by Vas2870 did not have any effect on either the total protein or phosphorylation levels of AMPK (*P* > 0.05, D8w + I/R + Vas2870 vs D8w + I/R). Similarly, in vitro in H9C2 cells, the total and phosphorylation levels of AMPK were substantially reduced post‐H/R in cells incubated with normal or high glucose (all *P* < 0.05, NG + H/R vs NG, HG + H/R vs NG + H/R). However, Nox2 gene knockdown did not influence the expression of AMPK or phospho‐AMPK (*P* > 0.05, HG + H/R + siRNA Nox2 vs HG + H/R). Nox2 levels were substantially heightened post‐H/R in both the normal and high glucose circumstances (*P* < 0.05, NG + H/R vs NG). After AMPK gene knockout, Nox2 levels were further increased (*P* < 0.05, HG + H/R + siRNA AMPK vs HG + H/R) and treatment with the AMPK agonist, AICAR, and decreased Nox2 protein levels (*P* < 0.05, HG + H/R + AICAR vs HG + H/R). These data suggest that AMPK and Nox2 interact with each other during myocardial I/R in diabetic hearts and that AMPK works upstream of Nox2.

### 3.5 AMPK attenuates post‐ischaemic myocardial injury and programmed cell death in diabetic rats

**FIGURE 5:**
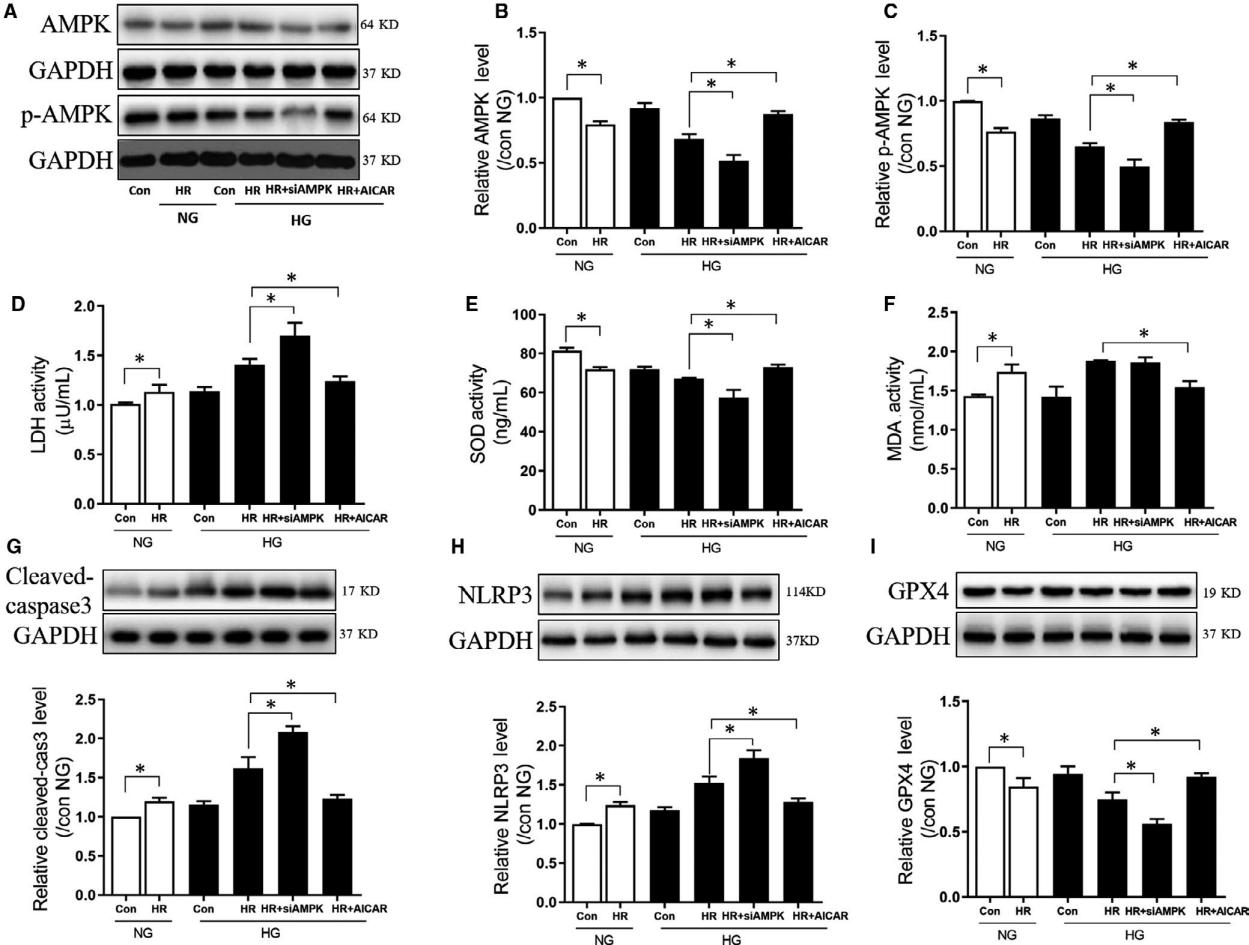
AMPK attenuated post‐ischaemic myocardial injury and programmed cell death in diabetic rats. A, WB bands depicting the protein expression of AMPK, p‐AMPKA and GAPDH; B, The change in protein expression of AMPK; C, The change in protein expression of p‐AMPK; D, LDH levels; E, SOD activity levels; F, MDA levels; G, The change in protein expression of cleaved caspase‐3; H, The change in protein expression of NLRP3; I, The change in protein expression of GPX4 during H/R in NG and HG, with or without siRNA AMPK, or agonist AICAR. In the HG group, H9C2 cells were subjected to 30 mmol/L glucose treatment for 48 h, H/R was achieved by 6 h hypoxia exposure and 12 h reperfusion. Data are expressed as mean ± SEM of two independent experiments, each performed in triplicates. n = 6 per group. *P < 0.05, **P < 0.01

AMPK has been demonstrated to defend diabetic hearts against myocardial I/RI by reducing cellular apoptosis. However, it is not clear whether AMPK has any effect on cell pyroptosis and ferroptosis during myocardial I/RI in diabetic hearts. As demonstrated in Figure 5B,C, in H9C2 cells, H/R decreased AMPK and p‐AMPK both under normal and high glucose situations (*P* < 0.05, HG + H/R vs HG, NG + H/R vs NG). The gene knockdown of AMPK further decreased AMPK and p‐AMPK expression (*P* < 0.05, HG + H/R + siRNA AMPK vs HG + H/R). Similarly, AICAR increased the decrement of AMPK and p‐AMPK induced by H/R (*P* < 0.05, HG + H/R + AICAR AMPK vs HG + H/R). Figure 5D shows that LDH release was drastically heightened post‐H/R under standard conditions (*P* < 0.05, NG + H/R vs NG). Gene knockdown of AMPK significantly increased post‐ischaemic cellular injury accompanied by increased LDH secretion (*P* < 0.05, HG + H/R + siRNA AMPK vs HG + H/R), while the AICAR significantly decreased LDH release (*P* < 0.05, HG + H/R + AICAR AMPK vs HG + H/R). Figure 5E,F shows that changes in oxidative stress during H/R including a decrease in SOD functioning and amplified MDA levels under normal condition (*P* < 0.05, NG + H/R vs NG). The gene knockdown of AMPK further decreased SOD activity (*P* < 0.05, HG + H/R + siRNA AMPK vs HG + H/R), but had no effect on the MDA levels (*P* > 0.05, HG + H/R + siRNA AMPK vs HG + H/R). AICAR treatment reversed the changes of oxidative stress induced by H/R (*P* < 0.05, HG + H/R + AICAR AMPK vs HG + H/R). Apoptosis, pyroptosis and ferroptosis were all significantly increased during H/R under normal condition, as indicated by increased levels of cleaved caspase‐3 and NLRP3 (*P* < 0.05, NG + H/R vs NG), and decreased expression of GPX4 (*P* < 0.05, NG + H/R vs NG). The gene knockdown of AMPK further enhanced these changes (*P* < 0.05, HG + H/R vs HG, HG + H/R + siRNA AMPK vs HG + H/R), and AICAR treatment significantly decreased cell death‐related changes induced by H/R (*P* < 0.05, HG + H/R + AICAR AMPK vs HG + H/R). In H9C2 cells that were administered HG and H/R, induction of AMPK attenuated post‐hypoxic cellular injury, reduced oxidative stress and decreased the induction of apoptosis, pyroptosis and ferroptosis. These data indicated that AMPK conferred cardio‐protection in the diabetic hearts by reducing oxidative stress and programmed cell death.

## 4 DISCUSSION

Our study shows that diabetes aggravates myocardial I/RI by activating the NADPH oxidase pathway in vivo and in vitro. Nox2 suppression was correlated with smaller IS, reduced oxidative stress and stimulation of myocardial apoptosis, pyroptosis and ferroptosis after I/R or H/R injury. Taken together, this study indicates that Nox2 contributes to diabetic myocardial vulnerability during I/RI. Furthermore, the underlying mechanism of diabetic‐related Nox2 activation may be mediated through suppression of AMPK throughout myocardial I/RI. Activation of AMPK in vitro can directly lead to the inhibition of myocardial Nox2 expression, a decrease in oxidative stress and subsequent programmed cell death.

Diabetes, a worldwide issue, makes it more difficult for patients with myocardial infarction to recover heart function after undergoing procedures, such as percutaneous transluminal coronary intervention (PCI) due to increased myocardial I/R vulnerability caused by diabetes.^17^ ROS, which is generated from the NADPH oxidase pathways, have a vital function in I/R damage in the diabetic heart.^18^ The excessive generation of ROS leads to lipid peroxidation‐mediated injury of the cell membrane, which accelerates programmed cell death.^19^ However, Nox2 is one of the most important subunits of the NADPH oxidase system, but its function in the diabetic myocardial I/RI is not yet known. In this study, we show that myocardial Nox2 activation occurs specifically in myocardial tissue after myocardial I/RI in diabetic mice. In comparison with healthy mice exposed to myocardial I/R, diabetic mice exhibited higher IS and programmed cell death, suggesting enhanced I/RI. Furthermore, oxidative stress significantly worsened injury to diabetic heart following I/RI. These changes were suppressed by administration of NAC (NADPH oxidase inhibitor) or Vas2870 (Nox2 inhibitor) in vivo, indicating a vital role for Nox2 activation in enhanced cardiac I/R injury in diabetic.

Although the vulnerability of diabetic myocardium to ischaemic injury is still controversial, it is an indisputable fact that the patients with diabetes are more likely to develop heart failure and have higher mortality after myocardial infarction.^20^ In diabetic condition, the AMPK pathway is highly correlated with myocardial I/RI.^21^, ^22^ Recently, studies showed that AMPK activity also had closed relationship with the smooth muscle, adipose tissue and liver tissues in diabetic animal.^16^, ^23^ These data are comparable to our findings in this study, which indicated that AMPK levels were substantially reduced in myocardial tissue from models of streptozotocin‐induced diabetes mellitus.^24^ Our results are similar to those reported by Ko et al^23^ who had found that insulin‐deficient mice also showed reduced myocardial AMPK activation after streptozotocin administration. AMPK activity has also been shown to be inhibited in the hearts of mice fed a high‐fat diet.^23^ Recently, AMPK has been identified as an energy sensor and a vital survival factor in the ischaemic heart.^25^ However, whether AMPK has a vital function in the diabetic myocardial I/R injury is still not clear. In this study, we show that treatment with AMPK agonist AICAR has beneficial effects in limiting myocardial I/RI by downregulating Nox2‐related ROS generation pathway in diabetic mice. However, we only show that AMPK inhibits Nox2 activation and downregulates downstream oxidative stress and programmed cell death. Whether or not AMPK directly or indirectly inhibits Nox2 is still unknown.

Recently, Balteau et al^26^ showed that in vitro glucagon‐like peptide 1 (GLP‐1) stimulated the α2‐isoform of AMPK and subsequently suppressed hyperglycemia‐stimulatedNox2 activation by limiting the protein kinase C (PKC)‐β2 phosphorylation andp47^phox^ activation. Whether AMPK suppresses Nox2 through GLP‐1 in diabetic hearts suffering from I/R injury still need to be further investigated. The cardioprotective effects in our study are similar to the ones reported by Kusmic et al^24^ and Balteau et al,^26^ who have shown that activation of AMPK by metformin or resveratrol can lead to a decrease in autophagy and cardiac microvascular function restoration in insulin‐deficient mice. Collectively, in light of previous studies, our study indicates that AMPK agonist could be an effective and promising drug in preventing and treating diabetic myocardial I/RI.

Of note, in this study, we show that diabetes can cause more severe myocardial I/RI by promoting myocardial cells to undergo three different types of programmed cell death including apoptosis, pyroptosis and ferroptosis. Apoptosis is not a passive process, but an active process, which involves the activation, expression and regulation of a series of genes. Apoptosis is not a phenomenon of self‐injury under pathological conditions, but a type of programmed cell death(PCD) process actively fought for to better adapt to the living environment.^27^, ^28^ Although the signalling pathways of apoptosis are different, the same apoptotic execution‐signalling pathway (activated by caspase family) induces the apoptosis in the end. Apoptosis has been identified as one of the main pathophysiological mechanisms of myocardial I/RI.^29^ The anti‐apoptotic intracellular signalling cascades which involved in myocardial protection can be reversed by diabetes.^30^ Pyroptosis, the inflammatory PCD mediated by inflammasome, is characterized by caspase‐1 dependence with the release of a large number of pro‐inflammatory cytokines.^31^ Damage‐associated molecular patterns were activated and increased expression of inflammasome components in the early stage of reperfusion. As the inflammasome gradually increase and reach the threshold for activation, inflammasome rapidly increases myocardial infarction via pyroptosis. Pyroptosis occurs more rapidly than apoptosis and is accompanied by the release of a large number of pro‐inflammatory cytokines.^32^, ^33^ Ferroptosis occurs during myocardial I/RI for the accumulation of lipid peroxidation products. Ferroptosis is an iron‐dependent PCD that is distinct from apoptosis and other types of PCD, characterized by accumulation of intracellular reactive oxygen species, which is inhibited by iron chelating agents and antagonized by such as ferrostatin‐1 and liproxstatin‐1, while apoptosis inhibitors (z‐vad‐fmk) and necrostatin‐1 have no effects on ferroptosis.^34^, ^35^

Recently, the interaction between different types of PCD is the focus of current research. For example, caspase‐3 has long been regarded as the hallmark of apoptosis, but Wang et al^32^ reported that the caspases cleave gasdermin D (GSDME) is cleaved and activated specifically by caspase‐3 and this cleavage causes pyroptosis. This suggests that there is a correlation between different forms of PCD, and in some cases, a specific stimulus causes another form of PCD. However, in the present study, our results showed that ischaemia reperfusion just like a ‘master key’ that can cause multiple forms of PCD including apoptosis, pyrotosis and ferroptosis. However, whether there is a crosstalk between these PCD forms remains to be further studied.

The hall marker of diabetes is hyperglycaemia, and hyperglycaemia‐induced ROS contributes to the myocardial injury. In diabetes, ROS was not only induced by hyperglycaemia but also can be induced from damage mitochondria. Based on this, it seems to be that ROS was the major detrimental factor to myocardial I/RI in diabetes. Since the ferroptosis was mainly ROS and iron‐dependent, and iron was one of the fundamental elements of ROS sources, and ferroptosis may be the first cell death in the process of diabetic myocardial.^4^, ^36^ However, different kinds of cell death have interactions and once one cell death occurs, other cell death could be stimulated. In future study, to explore the further and clear mechanism of different cell death during diabetic heart will provide potential treatment target for diabetes. Up to now, it is not known which form of cell death has a dominant function in diabetic myocardial I/R damage. Additional studies are required to examine the three types of programmed cell death in vivo and in vitro to clarify their roles in the progress of diabetic myocardial I/RI.

In conclusion, diabetes worsens myocardial I/RI by activating the Nox2‐related oxidative stress generation in an AMPK‐dependent manner, which subsequently induces different types of programmed cell death including apoptosis, pyroptosis and ferroptosis.

## CONFLICT OF INTEREST

The authors declare no conflicts of interest.

## AUTHOR CONTRIBUTIONS

Zhongjun Zhang and Weifeng Yao conceived and designed the experiments. Chunyan Wang, Lijie Zhu, Wenlin Yuan and Lingbin Sun performed the experiments. Chunyan Wang analysed the data. Zhengyuan Xia contributed reagents, materials and analysis tools. Chunyan Wang and Weifeng Yao wrote the paper. All authors read and approved the manuscript.

## Data Availability

The datasets generated during and/or analysed during the current study are available from the corresponding author on reasonable request.
